# The choroid plexus as a site of damage in hemorrhagic and ischemic stroke and its role in responding to injury

**DOI:** 10.1186/s12987-017-0056-3

**Published:** 2017-03-28

**Authors:** Jianming Xiang, Lisa J. Routhe, D. Andrew Wilkinson, Ya Hua, Torben Moos, Guohua Xi, Richard F. Keep

**Affiliations:** 1 Department of Neurosurgery, University of Michigan, R5018 BSRB, 109 Zina Pitcher Place, Ann Arbor, MI 48109-2200 USA; 2 Department of Health Science and Technology, Aalborg University, Aalborg, Denmark; 3 Department of Molecular & Integrative Physiology, University of Michigan, Ann Arbor, USA

**Keywords:** Intraventricular hemorrhage, Subarachnoid hemorrhage, Intracerebral hemorrhage, Cerebral ischemia, Choroid plexus, Cerebrospinal fluid, Blood–brain barrier

## Abstract

While the impact of hemorrhagic and ischemic strokes on the blood–brain barrier has been extensively studied, the impact of these types of stroke on the choroid plexus, site of the blood-CSF barrier, has received much less attention. The purpose of this review is to examine evidence of choroid plexus injury in clinical and preclinical studies of intraventricular hemorrhage, subarachnoid hemorrhage, intracerebral hemorrhage and ischemic stroke. It then discusses evidence that the choroid plexuses are important in the response to brain injury, with potential roles in limiting damage. The overall aim of the review is to highlight deficiencies in our knowledge on the impact of hemorrhagic and ischemic strokes on the choroid plexus, particularly with reference to intraventricular hemorrhage, and to suggest that a greater understanding of the response of the choroid plexus to stroke may open new avenues for brain protection.

## Background

The choroid plexuses (CPs) are present in the lateral, third and fourth ventricles of the brain (LVCP, 3rd CP, 4th CP). Each CP is comprised of epithelial cells surrounding a richly vascularized core [1]. Compared to the cerebral capillaries that form the blood–brain barrier (BBB), CP capillaries are leakier with the endothelial cells having fenestrations, reflecting the role of the CPs in CSF secretion [2–6]. The CPs are the site of the blood-CSF barrier, with the CP epithelial cells being linked by tight junctions that limit paracellular diffusion [1, 7].

Many neurological conditions result in BBB dysfunction, including ischemic and hemorrhagic strokes [8–11]. That dysfunction participates in brain injury by, for example, causing vasogenic brain edema, allowing the entry of potentially neurotoxic compounds from blood to brain, and promoting leukocyte infiltration and neuroinflammation. In contrast to the wealth of studies on BBB injury, much less is known about whether CP injury occurs after stroke [12, 13]. One purpose of the present review is to discuss what is currently known about such injury. It addresses hemorrhagic and ischemic stroke, with a particular focus on the former including intraventricular hemorrhage (IVH), subarachnoid hemorrhage (SAH) and intracerebral hemorrhage (ICH).

Another purpose is to review a body of evidence that the CPs respond to injury (that may be distant from the CP) by releasing factors that protect the brain [14–16]. This has led to experimental studies with CP transplantation for a variety of conditions [17–19]. That section also addresses a related topic, namely the role of the CP in leukocyte entry into the brain after injury. Results on the protective effects of the CP in brain injury/disease may help to inform studies on the role of cerebral endothelial cells.

## Choroid plexus as a site of injury

This section discusses evidence of CP injury in different forms of stroke in humans and animals. In human studies, it discusses morphological (post-mortem), imaging and permeability data to assess injury. One advantage of studies on the CP compared to the BBB is that the CSF (unlike the brain) can be sampled in patients, although it should be noted that CSF composition can reflect changes at both the blood-CSF and blood–brain barriers. In animal studies, the CP itself, as well as the CSF, can be sampled. Inflammatory changes will be discussed in the next section (CP as a responder to injury). Additionally, this section examines whether hemorrhage (particularly bleeding within the ventricles) can cause CP injury. It does not discuss the CP being the site of initial bleeding, although some evidence indicates CP hemorrhage is a major cause of IVH in babies born at full term [20] and can occur at other ages [21, 22].

### Intraventricular hemorrhage

IVH commonly occurs in premature infants as a result of germinal matrix hemorrhage. It is a major cause of cerebral palsy in such infants and is often associated with later development of hydrocephalus [23]. In adults, intraventricular extension of bleeding occurs in ~50% of patients with ICH and ~45% of patients with SAH [24, 25]. Such ventricular extension is a risk factor for poor outcome after both ICH and SAH [25–27]. ICH is known to cause perihematomal tissue damage [28] and BBB dysfunction [10]. Clot-derived factors, including hemoglobin, iron and thrombin, play a major role in ICH-induced injury [28]. Despite this evidence in ICH and the proximity of intraventricular blood to the CPs, we did not find clinical studies examining the effects of IVH on the CP epithelial morphology in the literature. Clinically, IVH results in changes in CSF protein concentrations (e.g. [29–33]). While changes in blood-CSF barrier function are one parameter that can affect those concentrations, assessing barrier function from such data is very difficult, particularly in the setting of IVH. Thus, for example, total CSF protein is elevated after IVH [29], but this may reflect protein originating from the initial hemorrhage, protein influx at the BBB as well as the blood-CSF barrier and production from brain parenchyma. Examining the CSF/plasma ratio of specific proteins, such as albumin [30, 32], helps address the latter point, but albumin will still enter CSF from the initial IVH as well as any potential BBB disruption.

In animals, Simard et al. [34] examined a rat IVH model at 2 days and found induction of inflammatory signaling (nuclear factor κB activation) within the CP along with a significant intracellular uptake of IgG. Furthermore, Gram et al. [35] found a marked inflammatory response in a rabbit preterm model of IVH at 1 and 3 days. This was accompanied by CP cell death, caspase activation and pronounced ultrastructural changes. Similar inductions in inflammatory mediators and cell death were obtained in vitro when CP epithelial cells were exposed to met-hemoglobin, heme or CSF from preterm babies with IVH [35]. The hemoglobin scavenger, haptoglobin, prevented the adverse effects of IVH in vivo and hemoglobin or IVH CSF in vitro [35].

**Fig. 1:**
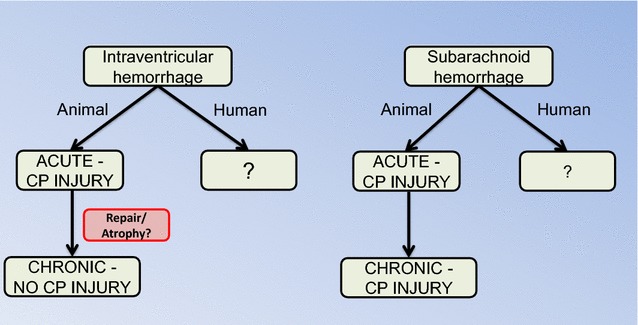
In general, there is a surprising lack of data on the effects of intraventricular and subarachnoid hemorrhage on choroid plexus (CP) injury, particularly in humans. In intraventricular hemorrhage, there are studies indicating acute CP damage but this may be absent in the long-term. This could reflect repair mechanisms (see Fig. 2 for cerebral ischemia) and there is a need for longitudinal studies in the same model. For subarachnoid hemorrhage, there is evidence for both acute and chronic CP injury

In contrast, Pang et al. [36] found only subtle changes in CP morphology at 3 months in a dog IVH model. In comparison, the ependymal lining of the lateral ventricles was destroyed or interrupted in many areas. Interestingly, they also examined the effects of urokinase in their model to lyse the IVH and found that this protected the ependyma but also resulted in CP atrophy in some animals for unknown reasons. While it is possible that there may be species differences in the effects of IVH on the CP, a more likely reason for the differences between the studies are the time points examined. As described below, in SAH and ischemia there is evidence of acute injury followed by a recovery in CP structure, although the latter may be accompanied by a reduction in CP size. Acute studies (1–3 days) in larger species and chronic studies in rodents would be informative (Fig. 1).

As opposed to the paucity of data on CP injury in human IVH or animal models, there is considerable data on damage to the ependymal cells that line the cerebral ventricles. Thus, Fukumizu et al. [37, 38] found marked ependymal damage and loss in human neonatal IVH. Mayfrank et al. [39] used a pig IVH model with and without tissue plasminogen activator-induced fibrinolysis and, similar to Pang et al. [36] in dog, they found marked IVH-induced ependymal damage at 1 and 7 weeks and that was reduced by fibrinolysis. In rat, Simard et al. [34] found that ependymal cells took up IgG at 48 h post IVH and Gao et al. [40] found ependymal damage 1 day after intracerebroventricular (ICV) injection of Fe^3+^, a degradation product of hemoglobin. These results indicate that there can be acute and chronic ependymal damage after IVH. In the CP after ischemic injury, there is evidence of tissue repair with time (see below). Why this does not happen in the ependyma is uncertain but it might relate to the ability of the CP to shrink in size when shedding dead cells. Because of the physical contact between the ependyma and underlying brain, such shrinkage is not possible for the ependyma.

### Subarachnoid hemorrhage

SAH is often associated with extension of blood into the ventricular system (30–50%; [25, 27]). IVH is a risk factor for poor prognosis in SAH and for the development of hydrocephalus and vasospasm [25, 27, 41]. Rosen et al. [25] found that hydrocephalus developed in 62% of SAH patients with IVH compared to 26% of patients without IVH. Post-hemorrhagic hydrocephalus is generally assumed to result from a block in the CSF flow pathways or fibrosis at CSF outflow sites [42, 43]. Whether SAH (with or without associated IVH) causes CP injury has not to our knowledge been examined in patients. Given the preclinical data indicating that SAH causes CP damage in animals, this gap in knowledge needs to be corrected.

Several studies using cisterna magna blood injection in rabbits have reported effects on the CP (Fig. 1). Liszczak et al. [44] reported CP changes including electron-dense cytoplasmic inclusions and expansion of the lateral and subcellular spaces. Yilmaz et al. [45] examined both choroidal artery vasospasm and CP injury (using an injury scoring system) at 20 days and found evidence of a correlation between choroidal artery constriction and CP damage after SAH. In another study, the presence of water filled spaces within the CP was examined at 2 and 14 days after SAH [46]. There was a marked increase in the numbers of such spaces, particularly at 2 days and the authors hypothesized that these changes may be related to altered CSF secretion. Furthermore, changes in CP morphology, with reduced epithelial cell and microvillus height, and many TUNEL-positive cells have been reported 10 days after SAH (24% of cells in animals surviving SAH and 42% in animals that died) [47].

As in humans, animal models of SAH can also cause hydrocephalus. Thus, using a endovascular perforation model of SAH (perforation at the internal carotid artery bifurcation), Okobu et al. [48] found evidence of acute hydrocephalus in about 40% in rats. A question that has not been totally resolved is whether there has to be blood in the ventricles to induce CP injury and hydrocephalus, although Okobu et al. [48] found that hydrocephalus was always associated with blood in the ventricular system.

### Intracerebral hemorrhage

Intraventricular extension of bleeding is a common finding in ICH and a risk factor for poor outcome [24, 26]. Whether ICH without such IVH can impact CP function has not been examined. Even though there is no extension of blood into the ventricular system, products from clot degradation may move into the CSF [49].

### Ischemic stroke

**Fig. 2:**
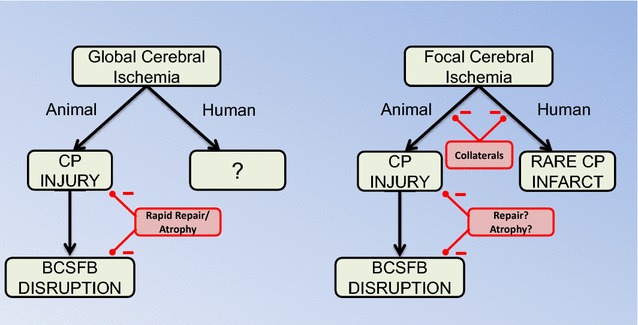
In animal studies, there is evidence that both transient global cerebral ischemia and focal cerebral ischemia cause choroid plexus (CP) injury and blood-CSF barrier (BSCFB) disruption. There is evidence that the damage after global ischemia is quickly repaired and this probably involves shedding of damaged cells resulting in CP atrophy. This has not been examined in focal cerebral ischemia. In humans, the effects of transient global cerebral ischemia (e.g. cardiac arrest) on the CP have not been examined. There are very rare reports of CP infarcts after focal cerebral ischemia in patients, but the occurrence of CP damage after focal cerebral ischemia is almost certainly limited by the rich collateral circulation to the CP

In humans, the blood supply to the LVCP comes from the anterior, lateral posterior and medial posterior choroidal arteries [50]. The blood supply to the third ventricle CP comes from the bilateral medial posterior choroidal arteries [51], while that to the fourth ventricle CP is from the anterior and posterior inferior cerebellar arteries and the superior cerebellar artery [52]. The rich collateral circulation for each of the CPs may help protect them from focal ischemic events. Thus, using magnetic resonance imaging (MRI), only three CP infarcts have been described in the literature. Two related to choroidal artery occlusion [53, 54] and one due to a basilar artery occlusion [55]. Brain infarcts from anterior choroid artery (AChA) occlusion have been described by multiple groups (e.g. [56–59]). The AChA supplies multiple tissues (e.g. the internal capsule, optic tract and the cerebral peduncle; [58]) as well as the LVCPs. Whether changes in LVCP function occur and contribute to the neurological symptoms with AChA occlusion is uncertain. Infarcts related to the medial posterior choroidal arteries have rarely been described [54, 60] (Fig. 2). There have been few studies on the impact of transient global cerebral ischemia (e.g. cardiac arrest) on CP injury/function. However, in patients resuscitated after cardiac arrest, an increased expression of advanced glycation end-product receptors (RAGE) in CP epithelial cells and amyloid beta in both CP blood vessels and on the epithelial basement membrane have been reported [61, 62].

In animals, a number of studies using transient global ischemia models have shown CP epithelial cell death (Fig. 2). Thus, Pulsinelli et al. [63], using permanent bilateral basilar artery occlusion with 30 min transient common carotid artery occlusion (4VO model) in rats, found ischemic necrosis in the CP at 6 h and this is earlier than found in hippocampus suggesting selective vulnerability. Also, using the rat 4VO model, LVCP atrophy (~35%) [64] and histological damage [65] have been reported. Other studies have reported widespread epithelial cell death and CP edema early during reperfusion (0–12 h) after bilateral common carotid artery occlusion (10 min) with hypotension (2VO + hypotension) [66, 67]. Furthermore, CP DNA fragmentation occurred after 2VO + hypotension in rats at 18–24 h [68, 69]. In gerbils with 5 min bilateral common carotid artery occlusion with reperfusion, CP DNA fragmentation was found at ~1 week [70, 71].

In neonatal rats, Rothstein and Levinson [72] found that perinatal hypoxia/ischemia caused cell death in the CP and they concluded that the CP is selectively vulnerable. Likewise, Towfighi et al. [73] found pyknotic nuclei within the rat CP a few hours after hypoxia/ischemia and this progressed to necrosis by 24 h. Interestingly, similar to the adult, those necrotic cells disappeared within 4 days of the CP becoming atrophied. Sivakumar et al. [74] examined the effects of 2 h of hypoxia alone in neonatal rats and found CP morphological signs of injury at 3 and 24 h. These began to resolve at 3 days with the CPs having a normal appearance at 14 days. It should be noted that one study in neonatal mice reported no damage to the CP after hypoxia/ischemia injury [75]. It is interesting, however, that many of these studies report a recovery in CP structure and loss of damaged cells with time [63, 64, 66–69, 73]. This recovery may reflect the loss of damaged cells, the recruitment of new cells or both. The CP atrophy reported by Dienel [64] and Towfighi et al. [73] suggests the former, but there is some evidence for cell recruitment (see below). In terms of blood-CSF barrier function, barrier disruption was reported by Ikeda et al. [70] after bilateral common carotid occlusion in the gerbil. Similarly, Ennis and Keep [76] reported blood-CSF barrier disruption at 6 h after 2VO + hypotension in the rat.

There have been few studies on CP injury after focal ischemia [76–79] (Fig. 2). It should be noted that the commonly used suture model of middle cerebral artery (MCA) occlusion can result in reduced blood supply to the LVCP [76] as the branch point for the AChA from the internal carotid artery is close to the bifurcation of the MCA [80]. In the rat, Ennis and Keep [76] found a 38% decrease in LVCP blood flow after MCA occlusion and there was a 53% reduction if the animals underwent a tandem common carotid artery occlusion. Nagahiro et al. [79] examined the effects of different durations of tandem MCA and internal carotid occlusion in rats. They found evidence of blood-CSF barrier disruption with MRI using gadolinium-diethylene triamine pentaacetic acid (Gd-DTPA) in animals with 15 and 30 min of ischemia with 6 h of reperfusion, whereas BBB disruption was only found with longer (60 min) periods of ischemia. They did not find evidence of CP morphological changes after 6 h of reperfusion by H&E staining. Li et al. [78], studying 2 h transient MCA occlusion and reperfusion in rats, reported that CP cells became swollen (1–4 days) and that there was bromodeoxyuridine (BrdU) staining indicating cell proliferation, perhaps in compensation for CP cell death. No BrdU staining was observed in the CP in non-ischemic rats. Interestingly, the BrdU staining after MCA occlusion was not limited to the LVCP, but also occurred in the 3rd and 4th CPs indicating effects on proliferation distant to the ischemic parenchyma. Gillardon et al. [77] found evidence of LVCP epithelial cell death (TUNEL staining) after 6 h but not 1.5 h of permanent MCA occlusion in the rat and Ennis and Keep [76] found evidence of LVCP edema after MCA occlusion in rats with and without tandem common artery occlusion.

A number of co-morbidities increase the risk of ischemic stroke and result in more severe brain injury after stroke. These include aging, hypertension, hyperlipidemia and hyperglycemia and they are known to enhance BBB dysfunction after stroke [81]. The effects of such co-morbidities on CP injury after ischemic stroke are unknown. However, even in the absence of stroke, it is known that aging and Alzheimer’s disease result in CP dysfunction [5, 82–84].

## Choroid plexus as a responder to injury

**Fig. 3:**
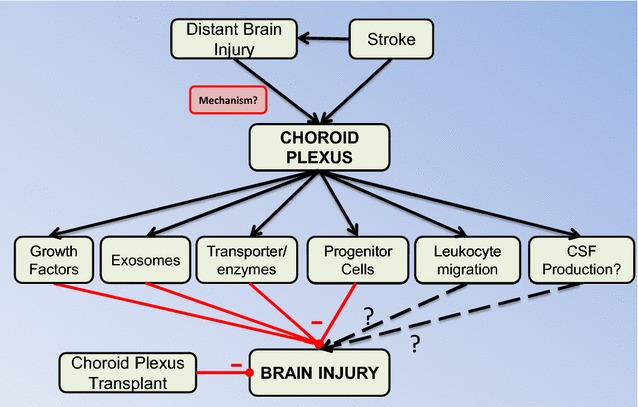
As well as being a potential site of injury after stroke, the choroid plexus (CP) also responds to stroke in multiple ways that may modify brain injury. It should be noted that the potential protective CP responses may be elicited by injury distant from the CP, indicating an unknown signaling mechanism. While most of the CP responses are thought to be protective, the effects of leukocyte diapedesis and changes in CSF production (if any) are uncertain. Elucidation of protective mechanisms involving the CP led to studies where CPs or CP epithelial cell transplants were used in animals with ischemic stroke with evidence of neuroprotection

As well as being a site of injury, the CP may be involved in a neuroprotective response after brain injury. This includes CP secretion of protective factors, the CP as a site of neurogenesis/progenitor cell migration, the CP as a site of leukocyte infiltration (although this may have beneficial and detrimental effects) and potential changes in CSF production (Fig. 3). The evidence on such roles is limited and, therefore, this section discusses brain injury/disease in general and not just stroke. It should be noted that responses can be to brain injuries that are distant to the CP raising the question of how those injuries are ‘sensed’.

### Growth factor/neuropeptide release

The CPs produce a wide range of peptides/proteins: e.g. adrenomedullin, basic fibroblast growth factor (bFGF)-2, insulin-like growth factor (IGF)-2, transthyretin, transforming growth factor (TGF) β and vasopressin, many of which are secreted into the CSF [14–16]. There they can impact periventricular cells but they may also have an autocrine function since the CPs also possess an array of receptors: e.g. the FGFR1 and FGFR2 for fibroblast growth factor, IGF-1R and IGF-2R for insulin-like growth factor, TβRII for TGFβ, and the vasopressin receptors V_1a_, V_1b_ and, during development, V_2_ [14]. There is evidence of CP-induced neuroprotection via growth factor release in a number of injury and disease states including stroke (see below) and traumatic brain injury [15, 85]. It should be noted, however, that some peptide release by the CP can have adverse effects. Thus, for example, there is evidence that vasopressin exacerbates brain injury after stroke [14].

With respect to animal models of stroke, studies have focused on cerebral ischemia and not the CP response to cerebral hemorrhage. Knuckey et al. [86] found that TGFβ-1, -2 and -3 mRNA expression in the CP increased at day 1 and 2 after transient global ischemia (2VO + hypotension), returning to normal at day 3. Those changes in mRNA expression were not mirrored in altered CP protein expression and it was suggested that increased mRNA levels might reflect secretion into CSF. Increases in CSF TGFβ-1 have been reported in ischemic stroke and SAH [87, 88]. In cerebral ischemia, TGFβ-1 appears to be neuroprotective [89, 90] but it may have an adverse effect in IVH-induced and other forms of hydrocephalus [42, 91].

Hayamizu et al. [92] used immunohistochemistry to examine the effects of transient global cerebral ischemia (2VO + hypotension) on bFGF-2 expression in the CP and found that ischemia caused a marked decrease in cytoplasmic (but not apical or lateral membrane associated) expression from 6 h to 14 days post-ischemia. The loss of cytoplasmic staining was interpreted as reflecting CP to CSF secretion. Since ICV injection of bFGF-2 is protective in animal models of global ischemia [93, 94], this suggests that CP secretion has a protective role. Beilharz et al. [95] examined the response of IGF-1 and -2 and different IGF binding proteins (IGFBP) to hypoxia/ischemia in 3 weeks-old rats. They found that IGFBP-6 mRNA and protein was induced in the CP (as well as the ependymal and reactive glial cells) and hypothesized that IGFBP-6 binds particularly to IGF-2 limiting adverse effects of IGF-2 after hypoxia/ischemia. Sivakumar et al. [74] found increased VEGF mRNA and protein levels in the CP at different times after 2 h of hypoxia in neonatal rats. VEGF expression is regulated by hypoxia-inducible factor α and VEGF concentrations in CSF are increased by conditions causing brain hypoxia [96]. Additionally, there are multiple papers indicating a protective effect of ICV administered VEGF in ischemic stroke [97–100] suggesting that CP production of VEGF could have protective effects in stroke. However, it should be noted that Liu et al. [101] recently found that ICV administration of an anti-VEGF receptor-2 antibody reduced early brain injury in a mouse SAH model.

A concern with such growth factor studies is that using data on CP mRNA or protein expression to imply secretion into CSF could be misleading. It should be noted, however, that there is also in vitro data indicating such secretion. For example, Borlongan et al. [18] found that CP explants secrete glial-derived neurotrophic factor, brain-derived neurotrophic factor and nerve growth factor. Another concern is the relative importance of the CPs in secreting such factors into CSF after stroke compared to other brain areas. Data using selective inhibition of CP growth factor production during stroke would be very informative (e.g. CP specific inducible KO mice; [102]).

With respect to human stroke, Flood et al. [87] examined CSF TGFβ1 in patients who had hydrocephalus after SAH and found markedly increased CSF levels early and late after SAH. While the initial rise was probably related to platelet-derived TGFβ1, the authors found that late increase in CSF levels correlated with increased CP expression. Adrenomedullin is a vasoactive peptide that is secreted by the CP and other non-BBB areas of the brain. After SAH, there is an early (day 3) and particularly a late (day 8) increase in CSF adrenomedullin that does not correlate with plasma levels. Higher CSF levels were correlated with an increased risk of delayed ischemic neurological deficits, occurrence of hyponatremia and reduced appetite [103, 104]. Whether the elevated CSF adrenomedullin levels are due to increased CP secretion has not been directly examined.

Apart from secreting proteins directly into CSF, recent evidence indicates that CP epithelial cells also secrete exosomes into the CSF [105–107]. These contain proteins, microRNAs and other components and form another type of communications between CP and the brain parenchyma [105–107]. The role of exosomes in the protective effects of the CP in brain injury is still unclear.

### Choroid plexus enzymes and transporters

As well as secreting factors that may influence brain damage, the CPs also express many enzymes and transporters that may be important in the response to injury. Thus, unlike the CSF, the CP has high fibrinolytic activity [108, 109] and this may play a role in intraventricular hematoma resolution. In addition, considering the role that iron, as a degradation product of hemoglobin, has in brain injury after cerebral hemorrhage [28, 110, 111], it is noteworthy that the CP expresses a very wide array of iron-handling proteins including transporters: e.g. divalent metal transporter-1, ferroportin, transferrin and transferrin receptor-1 and -2, the iron storage protein ferritin, the heme metabolizing enzyme heme oxygenase-1, and proteins that change the oxidative sate of iron (ceruloplasmin and hephaestin) [112, 113]. The role of the CP in handling hemoglobin and iron after IVH merits further investigation. In relation to Alzheimer’s disease, the CP plays a role in regulating amyloid-β degradation and the levels of amyloid-β in the brain were reduced in a mouse model of Alzheimer’s disease by CP implants [17].

### Choroid plexus transplantation

Because of the evidence that the CP can protect the brain from injury, there have been a number of studies examining the effects of CP transplants on different types of brain injury. Borlongan et al. found that transplanting rat [114] or pig [18] choroid plexus into the CNS protected against cerebral ischemia in rats. Matsumoto et al. [115] also showed similar protection with cultured choroid plexus epithelial cells in rat MCA occlusion. Outside cerebral ischemia, Bolos et al. [17] found that CP epithelial cell transplants reduced amyloid-β deposits, tau hyperphosphorylation and astrocytic reactivity in a mouse model of Alzheimer’s disease. They also improved behavioral outcomes including memory tests. Similarly, transplants of CP epithelial cells have enhanced axonal regeneration and locomotor improvement in rat spinal cord injury [19, 116]. Compared to approaches that focus on a single factor (e.g. a growth factor) to promote brain protection/recovery, CP epithelial cells transplants may have an advantage in producing multiple factors. It is also possible that the CP epithelial cells may have a modified response dependent upon the type of injury. Thus, for example, will CP epithelial cells exposed to an ischemic brain produce the same growth factor and express the same enzymes as those exposed to a brain with Alzheimer’s disease?

### Choroid plexus proliferation and progenitor cells

In the adult CP, there is only a slow turnover of epithelial cells in rodents and primates [117, 118]. However, there is in vitro evidence that injury and growth factors (IGF and epidermal growth factor) can cause CP epithelial cell proliferation [119]. In vivo, Li et al. [78] found increased CP cell proliferation (BrdU positive cells) after MCA occlusion in the rat. The overall impact of such proliferation on CP injury in vivo is as yet uncertain (i.e. can it replace the cell loss caused by stroke?) and some of the BrdU cells were neuronal nuclear antigen (NeuN) and glial fibrillary acidic protein positive leading Li et al. to suggest they were neural precursor cells [78].

There has been considerable interest in the use of exogenous stem cells, either administered systemically or into the brain, to treat different neurological conditions. Endothelial progenitor cells administered intravenously can integrate into the cerebral endothelium after stroke and may be a source of growth factors [120]. Whether that is the case for the CP is unclear. Zhang et al. [121] found that bone marrow-derived endothelial progenitor cells migrated to the CP endothelium after intravenous injection in non-ischemic mice and Beck et al. [122] also found that bone marrow-derived cells given intravenously migrated to the CP in ischemic (MCA occlusion) and non-ischemic mice. They were uncertain whether those cells were integrating into the CP endothelium or migrating through the CP. However, mesenchymal stem cells given ICV can integrate into the CP epithelium [123, 124].

### Leukocyte migration at the choroid plexus

In stroke, neuroinflammation may have both detrimental and beneficial effects. Thus, an early influx of leukocytes (particularly neutrophils) into the brain from the blood may exacerbate ischemic and hemorrhagic brain injury [125, 126], but a later inflammation may be beneficial in brain repair [127–129]. In the case of cerebral hemorrhage, infiltrating macrophages/resident microglia have an important role in hematoma resolution via phagocytosis [130, 131]. Infiltrating leukocytes may enter the brain across the BBB or the blood-CSF barrier after a stroke. While there is a wealth of knowledge about leukocyte entry at the BBB in stroke, studies on the role of the CP are very limited. Kowarik et al. [132] compared CSF levels of leukocytes in patients across multiple neurological conditions. For stroke (a mixture of ischemic and hemorrhagic), they examined CSF within 4 weeks of onset. Unlike some conditions (e.g. bacterial or viral meningitis), stroke was not associated with high CSF leukocyte counts nor did it alter the distribution of leukocyte subsets. The majority of CSF leukocytes after stroke were CD4+ T cells (71%) and CD8+ T cells (17%). A fuller time course of such changes in CSF, particularly soon after stroke, would be informative.

The CP is thought to be involved in immune surveillance by trafficking T-lymphocytes [133, 134]. In a model of hypoxia/ischemia with systemic inflammation (lipopolysaccharide; to mimic intrauterine infection) in rats, Yang et al. [135] found an early (4 h) infiltration of T-lymphocytes (CD43+) into the CP. This was blocked by fingolimod (FTY720; a sphingosine-1-phosphate receptor modulator) treatment which also markedly reduced the brain injury in lipopolysaccharide-enhanced hypoxia/ischemia injury. In other neurological conditions, such as multiple sclerosis, traumatic brain injury, spinal cord injury and meningitis, as well as peripheral inflammation, there has been interest in the role of the CP in leukocyte entry into CSF and brain [3, 136, 137]. For example, Szmydynger-Chodobska et al. [138, 139] found that the CP is a site of neutrophil and monocyte entry into the brain after traumatic brain injury (controlled cortical impact). It is interesting that in those studies the CPs are distant from the site of initial injury raising the question as to what signaling mechanisms trigger the diapedesis of leukocytes at the CP.

### CSF production

Alterations in CSF production could impact brain injury. Increases could aid in the clearance of neurotoxic factors from the brain (CSF sink effect) but might also exacerbate hydrocephalus. With regards to the latter, choroid plexus hyperplasia and choroid plexus papillomas are associated with CSF hypersecretion and hydrocephalus [140]. Decreases in CSF production might serve to decrease the incidence of hydrocephalus and reduce intracranial pressure, and CP cauterization has been used as a treatment for hydrocephalus [141, 142]. After stroke, there is a lack of data on whether CSF production is changed. However, Holloway and Cassin [143] did report a 33% decline in CSF production with hypoxia in neonatal dogs.

The water channel, aquaporin 1 (Aqp1) is highly expressed in the CP epithelium and Aqp1 knockout mice have reduced CSF secretion [144]. There has, therefore, been an interest in examining the effects of stroke on Aqp1 expression. In the 4VO model of transient global cerebral ischemia, Akdemir et al. [65] reported an increase AQP1 protein expression in the CP epithelium between 24 and 48 h of reperfusion. Similarly, Sveinsdottir et al. [145] found an increase in CP Aqp1 protein expression in a preterm rabbit IVH model. However, at the mRNA level, there was a decrease in expression, an effect replicated in vitro by exposing cultured CP epithelial cells to the CSF of preterm infants with IVH or hemin. The reason for the different protein and mRNA responses are uncertain. Interestingly, Sveinsdottir et al. [145] also examined the expression of another aquaporin, Aqp5. They found a marked increase in both protein and mRNA expression in the preterm rabbit IVH model for that aquaporin and they also found increased Aqp5 mRNA after exposure of CP epithelial cells to hemin in vitro. This merits further investigation.

### Injury sensing by the choroid plexus

In the earlier descriptions of stroke-induced CP injury, this review has focused on types of injury that directly impact the CP, including reductions in CP blood flow or blood in the ventricular system adjacent to the CPs. However, there is evidence that events distant from the CPs may trigger protective responses in the CP. For example, traumatic brain injury that does not directly impact the CP results in growth factor production [15, 85], alters CP protein expression (e.g. [146]), and infiltration of leukocytes across the CP [138], and Li et al. [78] found increased cell proliferation in CPs distant to the parenchymal damage induced by a MCA occlusion. How does the CP sense that distant injury and what pathways in the CP are triggered to elicit the protective response? As noted above, the CP expresses a wide range of peptide/protein receptors [14] and these may be involved, but these are as yet largely unaddressed questions.

### Choroid plexus and cerebral endothelial responses to stroke

Our knowledge of the effects of stroke on cerebral endothelial cells, the site of the BBB, is much greater than at the CP epithelium. There are also differences in the types of experiment that can be performed in relation to each tissue. For example, with the cerebral endothelium, two-photon microscopy using fluorescently-tagged permeability markers and endothelial proteins (e.g. cells expressing GFP-tagged claudin-5) can be used to study stroke-induced BBB disruption in real time [147]. Such techniques can not currently be used to study the deeply-situated CPs. However, CP studies have an advantage in that CSF can be sampled (in animals and humans) in contrast to the cerebral endothelium studies where adjacent brain interstitial fluid is inaccessible. Thus, for example, the sampling of exosomes released from cerebral endothelial cells is currently limited to cultured cells [148]. While acknowledging that other sites (e.g. ependyma), as well as the CP, may contribute to CSF exosomes, analysis of such exosomes has given insight into exosome cargo in animals and humans in vivo (e.g. proteins, mRNAs and microRNAs [149–151]) as well as the impact of disease on exosomes [152].

Access to CSF has aided examination of the potential roles of the CP in protecting the brain from injury/disease. The results of such CP studies may aid in formulating brain endothelial studies. For example, to what extent will brain endothelial cells fulfil similar protective roles to the CP? While there has been considerable interest in the production of growth factors by neural and endothelial progenitor cells (endogenous and exogenous) after injury [153, 154], the role of the normal cerebral endothelium has received less attention.

## Conclusions

There have been surprisingly few studies examining the degree to which hemorrhagic stroke causes CP injury. This is particularly noticeable in the setting of IVH, where the CP is in close proximity to the hemorrhage. In ICH, there is considerable evidence that clot derived factors (e.g. hemoglobin and iron) cause perihematomal injury [28] and are a therapeutic target (intracerebral hemorrhage deferoxamine trial; iDEF—NCT02175225). Further clinical and preclinical studies on the extent to which IVH and SAH cause CP injury are needed as they may identify a therapeutic target.

Because of its rich collateral circulation, focal cerebral ischemia probably rarely causes CP infarction. However, there is evidence suggesting that the CP is selectively vulnerable to ischemia. Further studies on the impact of transient global ischemia (e.g. cardiac arrest) on CP injury in patients are warranted. The finding that the CPs can produce factors that can protect the brain from injury and disease raises many opportunities. The factors involved are probably numerous, making the effects difficult to reproduce pharmacologically (especially within the brain). Hence, there has been an interest in using CP transplants. However, there is a question as to what can trigger the release of protective factors from the CP? Determining the signaling pathway(s) may be a promising therapeutic avenue. It should also be noted that a role of the CP in neuroprotection after IVH or SAH has not really been examined and this may be an important area of research.
